# Li diffusion and migration are influenced differently by co-solvents in polymer electrolytes based on poly(ε-caprolactone) and poly(ethylene oxide)

**DOI:** 10.1039/d6sc00596a

**Published:** 2026-05-05

**Authors:** Simon Buyting, Monika Schönhoff

**Affiliations:** a Institute of Physical Chemistry, University of Münster Corrensstr. 28/30 48149 Münster Germany schoenho@uni-muenster.de; b International Graduate School for Battery Chemistry, Characterization, Analysis, Recycling and Application (BACCARA), University of Münster 48149 Münster Germany

## Abstract

In polymer electrolytes, poly(ε-caprolactone) (PCL) provides a lower lithium cation coordination strength as compared to the classically employed poly(ethylene oxide) (PEO), leading to enhanced lithium transference. Similar to PEO, however, PCL suffers from poor ionic conductivity, motivating plasticization with low-molecular weight co-solvents for improvement. To facilitate the choice of suitable co-solvents, we investigate here 15-crown-5 (15C5), tetra- and diglyme (G4, G2), dimethyl sulfoxide (DMSO), *N*,*N*-dimethyl formamide (DMF), sulfolane (SL), glycerol (GL) as well as propylene and vinylene carbonate (PC, VC) in terms of their effects on ion transport in PCL-based electrolytes with lithium bis(trifluoromethanesulfonyl)imide (LiTFSI) as a conducting salt ([Li^+^]/[monomer] = 0.25). Diffusion coefficients by pulsed-field gradient NMR and Raman analysis show a distinct difference between strongly and weakly Li-coordinating co-solvents, interestingly with a trend opposed to that for previously studied co-solvents in PEO-based electrolytes, which is explained by the competitive coordination of polymer and co-solvent to Li ions. Surprisingly, the strong influence of co-solvent coordination on diffusion does not result in an altered lithium transference number, *T*_+_, as obtained from ion mobilities determined by electrophoretic NMR. Ion migration in an electric field is far less dependent on the nature of the co-solvent, and even more, *T*_+_ is invariant between both polymers. We attribute this to the coupling of the species' hydrodynamic fluxes, which is dominating migration in an electric field, rendering *T*_+_ independent of polymer coordination and only weakly dependent on co-solvent coordination. Thus, our key conclusion is that while diffusive properties of ions are largely controlled by the interaction strength of polymer and co-solvent with Li^+^ ions, these mutual interactions hardly influence the migration in an electric field. Instead of coordination properties, it is hydrodynamic fluxes which couple the drift velocities during migration in an electric field, and ultimately dominate lithium transference. These findings also question diffusion coefficients as an indicator for Li transference.

## Abstract 2

Li-coordinating co-solvents have opposite influence on ion diffusion in PCL- and PEO-based polymer electrolytes, while ion migration remains unaffected.

## Graphical abstract

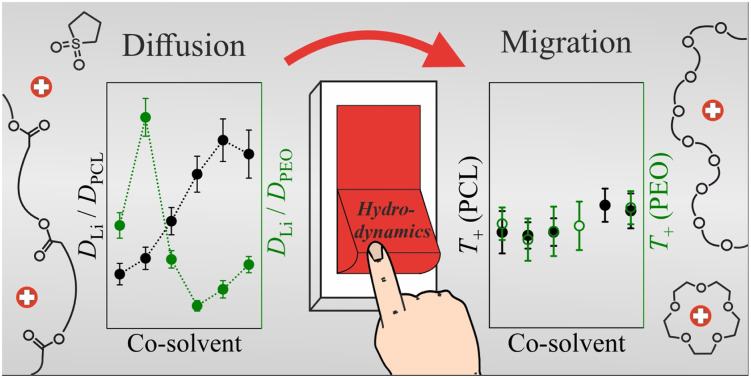

## Introduction

Only few other technologies have become as integral to modern life as the lithium-ion battery (LiB), which is nowadays the ultimate tool to flexibly store and retrieve electrical energy.^1^ However, in spite of its sophisticated design, mostly comprised of a carbon anode and a transition metal cathode, ionically connected by a carbonate-based, liquid electrolyte, especially the latter component leaves room for improvements.^2^ Although this liquid standard electrolyte offers fast lithium-ion conduction and therefore fast charging and discharging of the battery, it has several major shortcomings. These include its incompatibility with lithium metal, safety hazards due to leakage, flammability, volatility, toxicity and a poor mechanical stability.^3–5^ Polymer electrolytes are a way to overcome some of these limitations by replacing the liquid components by a mechanically stable, yet flexible polymer matrix. Extensive research in the past decades has shown that polymer electrolytes can not only represent a more environmentally friendly and safe alternative, but could also increase the energy density of the lithium-ion battery by switching from an intercalation material to a lithium metal anode.^6–9^

In order to introduce a polymer-based electrolyte to LiBs, the most decisive choice is the one of the polymer structure. Throughout the years, polymer architectures of seemingly ever-increasing complexity were investigated, including *e.g.* single-ion grafted side-chain copolymers,^10^ interpenetrating polymer networks^11^ and composite materials.^12^ However, a great deal of research on polymer electrolytes is still carried out on poly(ethylene oxide) (PEO),^13^ which was among the first polymers to be considered as LiB electrolyte material.^14^ Although PEO is much valued for providing strong dissociation of alkali metal salts, good processability and low glass transition temperature, its comparably low ionic conductivity at room temperature (RT) and its low lithium ion transference due to strong Li-polymer coordination was soon realized.^15,16^ These challenges were tried to tackle, *e.g.*, by using less strongly coordinating polymers such as polyesters and -carbonates, which can indeed yield higher lithium transference numbers.^17–20^ Among these, poly(ε-caprolactone) (PCL) represents a good compromise between a lower ionic conductivity due to a lower degree of dissociation and a higher lithium transference.^21^ However, similar to PEO, PCL also suffers from low RT conductivities due to its semicrystalline nature.^22^

One way to overcome limits imposed by low RT ionic conductivities is plasticization by low molecular weight co-solvents to form gel polymer electrolytes (GPE). While, in general, a large number of studies on plasticized polymer electrolytes is available, they rarely focus on the nature of the co-solvent, but rather make an empirical choice to increase ionic conduction, without systematically considering the influence of the co-solvent chemical structure and interactions.^23–25^ Only recently, a comprised study on diverse co-solvents in PEO-based electrolytes revealed their different effects on ion transport, especially depending on the ion coordination strength.^26^ However, as the performance of the co-solvent was shown to be intimately connected to their ability to coordinate lithium cations, the coordination strength of the polymer is expected to strongly influence the considerations on the choice of an appropriate co-solvent as well. Therefore, a study on the implications of co-solvent incorporation in more weakly coordinating polymer frameworks such as PCL is needed.

A valuable technique to study ion migration in electric fields, similar to the situation in a battery, is electrophoretic NMR (eNMR). Experimentally introduced and further developed by Holz and Johnson *et al.*, eNMR uses magnetic field gradients, in analogy to diffusion NMR, to monitor molecular transport, however, it combines them with the application of an electric field.^27,28^ The latter is applied by specially designed eNMR measuring probes, commonly consisting of palladium or platinum-black electrodes attached to different probe geometries ranging from U-tube designs to double cylinder and linear arrangements.^29^ While eNMR is nowadays a more established technique and major challenges like convection by Joule heating or electroosmosis were overcome by instrumental advancements, its application is still limited to a few groups worldwide.^30–35^ In particular, the successful measurement even of highly concentrated ionic liquids and polymer electrolytes represents an important cornerstone in recent eNMR advancements.^18,31,36^

In battery research, diffusion NMR by pulsed field gradients (PFG-NMR) is a widespread technique, delivering species-selective information about diffusive transport by ^7^Li, ^19^F and ^1^H PFG-NMR. However, the relevance of diffusion data for battery operation is limited, as the experimental set-up lacks an electric field. For example, transference numbers can only be estimated from diffusion coefficients under the assumption of an ideality and full salt dissociation, which is mostly invalid in concentrated electrolytes. Electrophoretic NMR, on the other hand, quantifies the ionic displacement in an electric field, which is applied in addition to a gradient echo, see Fig. S5. This yields the drift velocity, providing the electrophoretic mobility. This allows a model-free direct determination of partial conductivities and transference numbers.^37^ Moreover, eNMR recently enabled the elucidation of local volume conservation constraints as a governing factor for ion transport in battery electrolytes. In detail, this constraint implies that in an incompressible electrolyte the net volume flux, summed up over all migrating species, has to be zero, since density fluctuations are limited.^38^ While the universality of this constraint is immediately evident, velocity measurements by eNMR could shed light onto its significance for ion transport. One interesting consequence in polymer electrolytes is, *e.g.*, that due to the fast migration of large anionic species, a considerable, oppositely directed migration of non-charged species like polymer and co-solvents is induced.^26^ Furthermore, in PEO-based electrolytes, the increase in anion volume has even been shown to lead to an increase in lithium ion transference due to beneficial volume flux effects.^35^

Herein, we analyze both the diffusion and migration behavior in polymer electrolytes by means of pulsed-field gradient NMR (PFG-NMR) and eNMR to evaluate the consequences of the addition of diverse co-solvents on ion transport in PCL-based polymer electrolytes with lithium bis(trifluoromethane-sulfonyl)imide (LiTFSI) as a conducting salt. Raman spectroscopy serves to complement the data by an analysis of the degree of ionic association. By comparing co-solvents with different size and different coordination strength to lithium ions, dynamic and structural parameters are investigated. A contrast between diffusion and migration behavior is discussed in the light of volume conservation constraints. At last, since the range of investigated co-solvents largely agrees to the co-solvents investigated in a former study on PEO-based electrolytes,^26^ a direct comparison of both polymer frameworks and their different responses to co-solvent addition is drawn. This reveals a very interesting contrast in the influence of co-solvents on diffusion and migration in different polymer matrices.

## Experimental

### Materials

Lithium bis(trifluoromethanesulfonyl)imide (LiTFSI, Sigma-Aldrich, ≥99.0%) was dried overnight at 100 °C and 10^−6^ hPa while poly(ε-caprolactone) (PCL, Polymer Source Inc., *M*_n_ = 3500 g mol^−1^, PDI = 1.18, OEt and OH-capped) was used as received. Co-solvents 15-crown-5 (15C5, 98%), tetraethylene glycol dimethyl ether (G4, ≥99.5%), diethylene glycol dimethyl ether (G2, 99.5%), *N*,*N*-dimethyl formamide (DMF, 99.99%), sulfolane (SL, 99%), 1,1,3,3-tetramethoxypropane (TMP, 99%), propylene carbonate (PC, 99.7%), glycerol (GL, >99.5%) and vinylene carbonate (VC, >99%) were obtained from Sigma-Aldrich (Darmstadt, Germany), while dimethyl sulfoxide (DMSO, 99.6%) was obtained from VWR Chem (Darmstadt, Germany). All co-solvents were dried over molecular sieves to remove trace amounts of water. Electrolytes were prepared by mixing Li salt and PCL in a ratio of four monomeric units of PCL per salt molecule (*r* = [Li]/[monomer] = 0.25) at a maximum temperature of 120 °C until a homogenous solution was obtained. For the systems with co-solvent (CS), one of the above compounds was added in a molar ratio of [CS]/[Li] = 1.635 and the mixture was again stirred at a lower temperature (maximum 90 °C depending on co-solvent). Sample preparation and storage was done in an argon-filled glovebox.

### Impedance spectroscopy

Conductivities were measured employing an Alpha-A Frequency Analyzer (Novocontrol, Montabaur, Germany) with an alternating current voltage of 10 mV of frequency 10^−1^ to 10^7^ Hz. Samples were filled in TSC70 sample cells equipped with two polished platinum electrodes in a PEEK housing and placed on a Microcell HC measuring stand (RHD Instruments, Darmstadt, Germany). A temperature of 90 °C was maintained by a 2416 temperature unit (Eurotherm, Limburg, Germany). Since no standard solution of defined conductivity at 90 °C is available, the cell constant *k* was determined by measuring the ionic liquid 1-butyl-1-methylpyrrolidinium bis(trifluoromethanesulfonyl)imide (Pyr_14_TFSI/BMPTFSI, Solvionic, 99.9%). The conductivity at 90 °C, needed to calculate *k*, was taken from two individually reported data sets from 0 °C to 80 °C and 100 °C by inter- or extrapolation,^39,40^ and independently confirmed in a coin cell. By extracting the resistance *R* from the high-frequency plateau of admittance *Y*′ = *R*^−1^, conductivities were calculated according to *σ* = *k*/*R* with an estimated instrumental error of 10%.

### Raman spectroscopy

Raman spectra of samples contained in NMR tubes were recorded on a MultiRAM spectrometer (Bruker, Rheinstetten, Germany) using an excitation laser wavelength of 1065 nm with a power of 263 mW and an aperture of 5 mm. The spectra of 0.5 cm^−1^ resolution were deconvoluted with the software OPUS 8.5 (Bruker). While band amplitudes, positions and widths were fitted to the individual spectra, a fixed shape of 60% Lorentz/40% Gauss as well as a general instrumental and fitting error of 5% were assumed.

### NMR methods

For NMR experiments, either an Avance Neo or Avance III HD 400 MHz spectrometer (Bruker, Rheinstetten, Germany) with a broadband gradient probe head (DiffBBO, Bruker) of maximum gradient strength 17 T/m was used. Experiments were performed on ^7^Li, ^19^F and ^1^H. Temperature was calibrated using a Pt100 thermocouple (Greisinger Electronics, Regenstauf, Germany) and maintained by a heated air stream. Exemplary NMR spectra can be seen in the SI, Fig. S1 and S2. Except for glycerol, all co-solvents provide ^1^H resonances, which are sufficiently separated from PCL resonances.

#### Diffusion

For diffusion experiments at 80 °C, samples were measured in tightly sealed 5 mm NMR tubes employing the Pulsed Field Gradient Stimulated Echo (STE) pulse sequence. Experiments on ^7^Li were also performed at 90 °C to allow calculation of correlation parameters from mobilities measured at 90 °C. Upon incrementally increasing the gradient strength *g* up to a maximum of *g*_max_ = 1.2 to 17 T m^−1^, depending on sample and nucleus, the decay in signal intensity *I* was fitted by the Stejskal–Tanner equation (eqn (1)) to obtain the diffusion coefficient *D*.^41^1
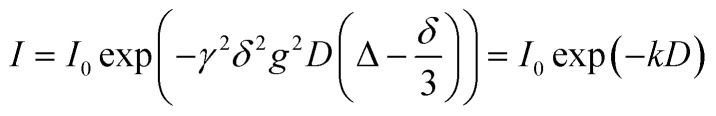
Here, *γ* is the gyromagnetic ratio. *A* gradient pulse duration *δ* of 1 to 3 ms and an observation time Δ of 100 to 300 ms were used, depending on sample and nucleus. Exemplary Stejskal–Tanner plots can be seen in Fig. S3. In each case, at least four different measurements were averaged and a general instrumental error of 5% is assumed.

For the polymer, non-linear echo decays were obtained as a consequence of its polydispersity (PDI = 1.18) and resulting diffusion coefficient distribution. Therefore, echo decays were fitted by a log-normal distribution of diffusion coefficients *P*(*D*) according to eqn (2) with P*(*D*) = *D* × *P*(*D*).2
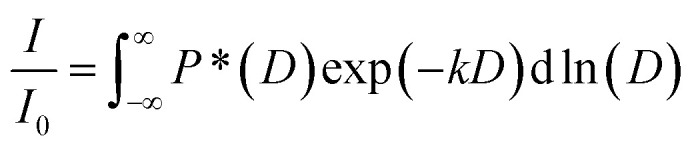
Here, *k* is defined by the experimental parameters, see eqn (1). Exemplary fitted echo decays of the polymer and the resulting diffusion coefficient distributions can be seen in Fig. S4. The maximum in *P*(*D*) was used for calculations and plots of PCL diffusion data.

Due to a significant overlap of resonances of glycerol and PCL in the ^1^H spectrum (cp. Fig. S1i and S2a), *D*_glycerol_ was obtained from the fast-decaying component of a simple biexponential fit. Here, the width of the underlying PCL diffusion coefficient distribution was neglected to limit the number of parameters, which is why a larger error of 10% was assumed.

#### Electrophoretic NMR

For electrophoretic NMR experiments, a self-built sample holder was used, which is described in detail elsewhere.^36^ Before each measurement, it was thoroughly cleaned and dried at 10^−6^ hPa and 90 °C for several hours to remove solvent and adsorbed water. Briefly, it consists of two palladium electrodes in a distance of 2.2 cm enclosed in a VESPEL^®^ rod, which routes the electric wiring, insulated by poly(tetrafluoroethylene) (PTFE) tubing, to a connection at the top. In between both electrodes, 60 polyimide-coated glass capillaries were placed to suppress convection in this region. The sample holder was then immersed into at least 350 µL of liquified sample. Remaining gas bubbles were removed by a prolonged storage at elevated temperatures and gentle tapping of the sample tube.

By applying a pulsed electric field during a diffusion NMR pulse sequence, a coherent drift of species by electrophoretic migration is induced, which results in a phase shift *ϕ* of the respective NMR signal:

3 *ϕ* = *γδg*Δ*Eµ*

Therefore, by incrementally increasing the electric field *E* = *U*/*d*, defined by the applied voltage *U* and electrode distance *d*, and analyzing the resulting phase shift, the electrophoretic mobility *µ* = *ν*/*E* can be obtained, which reflects the drift velocity *ν*.

In general, a double stimulated echo (DSTE) pulse sequence, accompanied by two pulsed electric field pulses of opposite polarity but same strength, was used for eNMR experiments (Fig. S5). The electric field was applied either by a self-built or a commercially available power source (eNMR 1000mc, P&L Scientific, Lidingö, Sweden). At that, a series of spectra was collected with the voltage incremented stepwise up to ±70 V for plasticized samples and ±100 V for samples without co-solvent, alternatingly starting with a negative or positive electric field. In general, an observation time Δ of 100 ms was used, which was only increased up to 200 ms where necessary due to very slow migration. The same applies to the gradient pulse duration, which was set to 1 ms and increased up to *δ* = 3 ms where necessary. The gradient strength *g* was fixed to a value between 0.6 and 8 T m^−1^ and a repetition time of 40 s was used to enable sufficient heat dissipation after electric field pulses. Phase shift analysis was done by fitting phase-sensitive Lorentzian profiles to the respective resonances using a self-written software, as described elsewhere.^42^^7^Li and ^19^F eNMR was employed for analyzing Li and TFSI mobility, and ^1^H eNMR served to determine CS and PCL mobility. In the latter case, resonances belonging to the same compound were jointly fitted with the same phase shift. To analyze the polymer mobility, separate experiments at higher gradient strength were performed, where the co-solvent resonance is suppressed due to diffusion. The exemplary spectra in Fig. S1 and S2 reveal a sufficient separation of solvent and polymer resonances to enable a stable fitting procedure. Exemplary phase shift plots of ^7^Li, ^19^F and ^1^H resonances are shown in Fig. S6. For each nucleus multiple measurements on at least 3 different sample fillings were performed to average the mobility and reduce the statistical error, while a general instrumental error of 10% is assumed. All eNMR experiments were performed at 90 °C and a comparison of ionic conductivity from eNMR, calculated by the partial conductivities (eqn (S1)) to ionic conductivities determined by impedance spectroscopy (Table S1), is performed to prove the data quality. Impedance and density data required for the comparison are given in SI, Section 3.1.

## Results & discussion

### Diffusion & interaction in PCL-based electrolytes

**Scheme 1:**
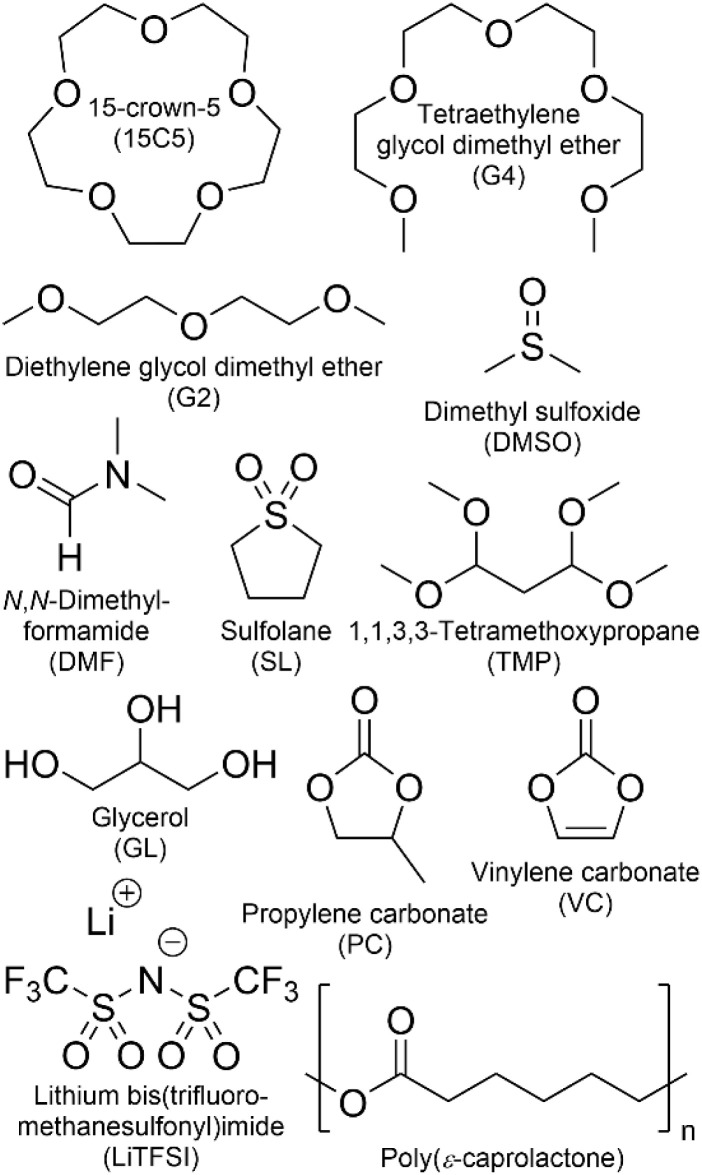
Investigated co-solvents, polymer and lithium salt.

To study the influence of varying co-solvents on ion transport in poly(ε-caprolactone) (PCL) polymer electrolytes, each of the co-solvents depicted in Scheme 1 was added to a mixture of PCL and LiTFSI in a ratio of 1.635 co-solvent molecules per lithium ion. The polymer-to-lithium ratio was chosen as such that 4 monomeric units of PCL are available per lithium cation. MD simulations had previously shown that Li^+^ is only coordinated by the carbonyl oxygen of the polyester.^43^ This implies that lithium cations are clearly undercoordinated by 4 monomeric units of PCL, since a preferred coordination number of 5 to 6 is reported.^43,44^ This specific composition was chosen (i) to promote an influence by solvent coordination, and (ii) to enable a comparison with the respective poly(ethylene oxide) (PEO)-based systems reported in a former study.^26^ Various co-solvents were selected to cover a wide range of different properties. As shown in Scheme 1, they include oligoethers, which are known to coordinate strongly to Li^+^ ions as well as carbonates, being commonly employed solvents in liquid battery electrolytes. In addition, the common polar solvents DMF and DMSO were employed, as well as sulfolane and glycerol, which showed beneficial properties in recent liquid electrolyte systems.^45,46^ This series covers a wide range of coordination strength to Li^+^ ions, as the binding constants in acetonitrile vary from the order of 10^4^ M^−1^ (15C5) to 10^−6^ M^−1^ (VC), as determined in earlier work.^26^

In this study, PCL of a low molecular weight of *M*_n_ = 3500 g mol^−1^ is used to ensure sufficient dynamics for diffusion and mobility studies, but the reported effects are expected to translate to systems of higher molecular weight, since the lower limit of entanglement was found to be around 2500 g mol^−1^ for PCL, which lies below the employed molar mass.^47^

**Fig. 1:**
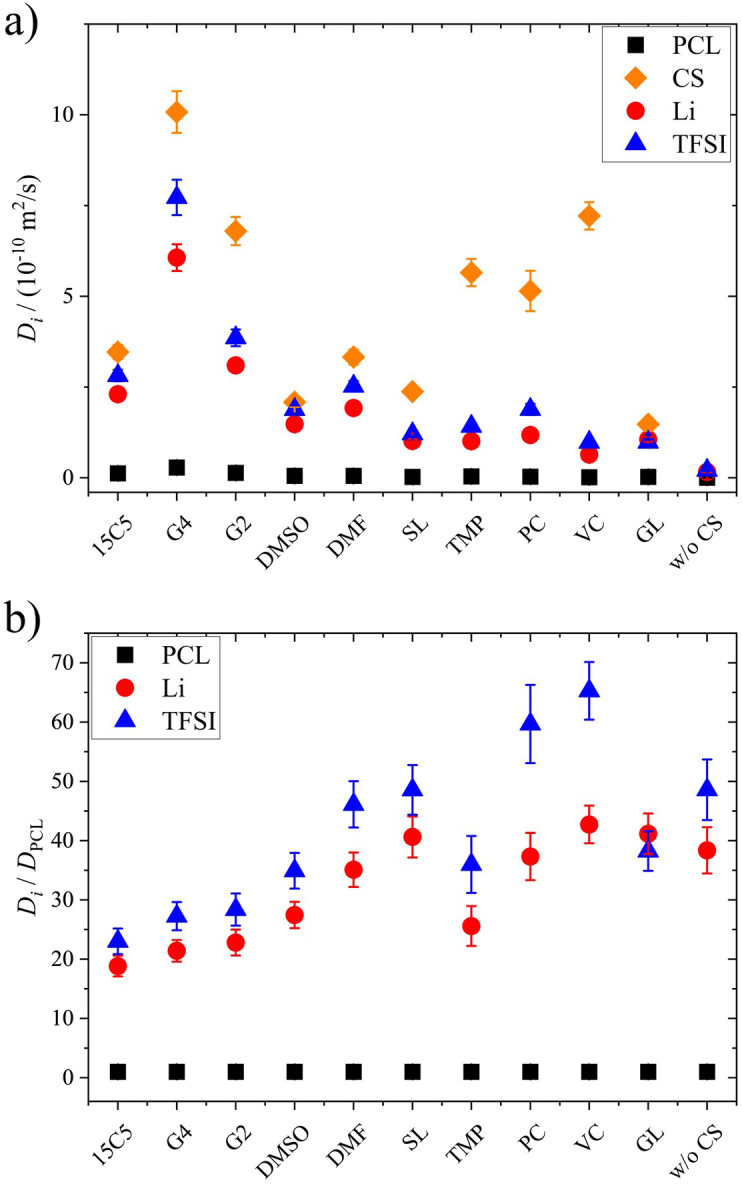
Diffusion coefficients *D* at 80 °C obtained by PFG-NMR of PCL + LiTFSI electrolytes containing different co-solvents and of the neat electrolyte: (a) original data, (b) data normalized to the polymer diffusion. The co-solvent coordination strength to lithium cations, except for glycerol, decreases from left to right.

To elucidate the effect of co-solvents on ion transport, diffusion coefficients were measured for the neat and plasticized polymer electrolytes. Diffusion echo decays for all species in exemplary samples are given in Fig. S3 and S4, and the resulting diffusion coefficients in all samples are summarized in Table S2 and Fig. 1a. In these and all further plots, the co-solvents are ordered according to their coordination strength to lithium cations, which decreases from left to right. This order is based on binding constants determined by NMR titration in acetonitrile, which were reported in a former study, from which they are reproduced in Table S3.^26^ For glycerol, which is not soluble in acetonitrile no binding constant is available, therefore it is depicted as the last co-solvent. We note that the order of solvents by Li binding constant agrees well to the order of dielectric constant, but differs from the Gutmann number order.

As can be seen in Fig. 1a, except for glycerol, the order of diffusion coefficients, *i.e.* co-solvent > TFSI > Li > PCL is maintained for all electrolytes and reflects the expected behavior. While the polymer is intrinsically slow in its dynamics, the non-ionic, smaller co-solvents can diffuse rather freely. The fact that TFSI anions diffuse faster than lithium cations has been observed in other systems as well and reflects the more pronounced interactions of the cationic species with polymer and co-solvent.^48,49^ Only for glycerol, lithium diffusion is slightly faster or equal to TFSI diffusion, which hints to a glycerol–anion interaction. Indeed, in binary systems of glycerol and lithium salts, higher diffusion coefficients were consistently measured for the cationic species and this finding was attributed to a strong hydrogen bond formation between anion and glycerol, leading to uncorrelated lithium dynamics.^46^

As a general effect of co-solvent addition, an increase in diffusion coefficients compared to the neat electrolyte is observed for all constituents, which is more pronounced in case of stronger coordinating co-solvents. However, as the most strongly coordinating co-solvents exhibit a higher molar mass than the weakly coordinating ones, in view of the constant molar fraction, part of this effect might be due to a higher mass fraction of strongly coordinating CS.

On the other hand, comparing diffusion coefficients in the 15C5 and G4 electrolytes, a large difference is observed, as the strong coordination by the crown ether in the form of [Li(15C5)]^+^ complexes is rather disadvantageous for diffusion. Tetraglyme, the open ring version of the crown ether, in contrast, leads to significantly higher diffusion coefficients, assumingly due to its higher flexibility and more diverse cation coordination structures in comparison to the crown ether. It is also interesting that very weakly coordinating co-solvents (TMP, PC and VC) appear to diffuse disproportionately fast in comparison to the remaining constituents, which indicates that Li coordination restricts co-solvent diffusion. In fact, a decreasing similarity of co-solvent and lithium diffusion coefficients can be observed in Fig. S7 upon a decreasing coordination strength.

**Fig. 2:**
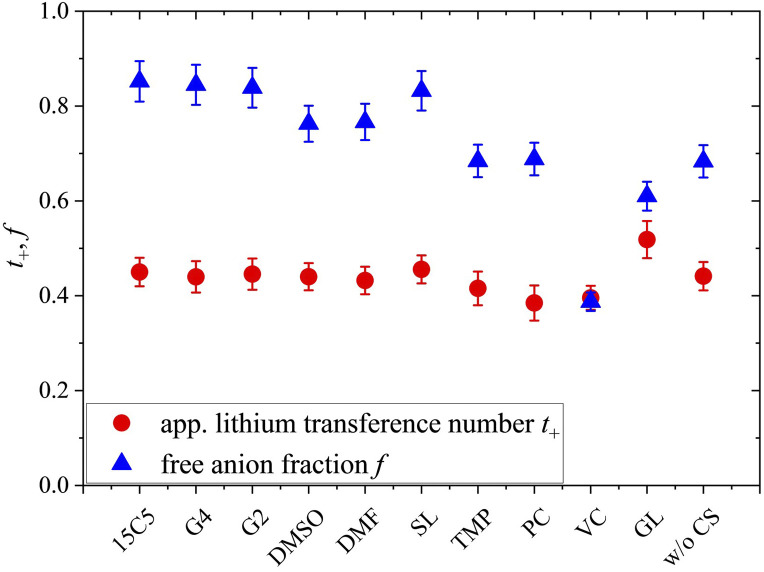
Apparent lithium transference number *t*_+_, estimated from diffusion coefficients at 80 °C, and free anion fraction *f*, analyzed by Raman spectroscopy. Error bars result from error propagation and an assumed instrumental error of 5%. The co-solvent coordination strength to lithium cations, except for glycerol, decreases from left to right.

Since the varying mass fraction of co-solvents could lead to a varying viscosity, affecting the diffusion coefficients, Fig. 1b shows the diffusion coefficients normalized to the polymer in order to roughly compensate viscosity influences. Remarkably, the normalized anion and cation diffusion coefficients clearly increase upon a decreasing co-solvent coordination strength to lithium cations. The large size of the strongly coordinating co-solvents 15C5 and G4 leads to a large Stokes radius of Li-CS coordination complexes. In the case of the weaker coordinating co-solvents the Stokes radius of any coordination complexes is smaller, or even more, the interaction is too weak for a transport in form of complexes. The anions appear to follow the behavior of the lithium cations. The smaller co-solvents SL, PC and VC, in contrast, cannot only diffuse more efficiently in the polymer network, as seen by high co-solvent diffusion coefficients in Fig. 1a, but they also do not form strong coordination complexes which could increase the Stokes radius of lithium cations. Further below we will discuss the influence of co-solvent molecular size and its disentanglement from coordination influences explicitly in context of the species volume fluxes. To closer examine the counterplay and association of anions and cations, Fig. 2 depicts the apparent lithium ion transference number *t*_+_, calculated from the diffusion coefficients according to eqn (4), see also numerical values in Table S3.4
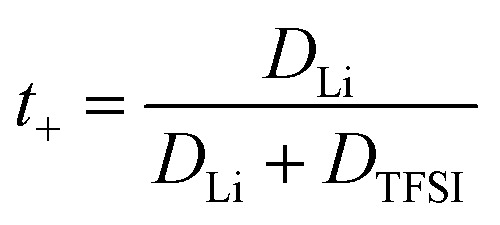


The free anion fraction, *f*, was analyzed by Raman spectroscopy using the area of Raman bands in the region of 740 to 750 cm^−1^, corresponding to the TFSI breathing mode.^50^ The Raman band of free (*i.e.* uncoordinated) anions at 742 cm^−1^ shifts to higher wavenumbers around 747 cm^−1^ upon the formation of a contact ion pair. Therefore, the ratio of free *versus* Li-coordinated anions can be determined by deconvoluting the respective Raman bands, provided that both species have a similar Raman scattering coefficient. The validity of this condition was shown by the agreement of different methods, which is why this form of analysis has found widespread application in electrolyte literature.^51–55^ Herein, the fraction of free anions was calculated by *f* = *A*_free_/(*A*_free_ + *A*_bound_), where *A* reflects the band area at the indexed wavelength. As can be seen in Fig. S8, all Raman spectra are well represented by the assumption of two separate bands. Details of the deconvolution parameters and the numerical free anion fractions are given in Table S4.

As can be seen in Fig. 2, the free anion fraction is slightly lower for weakly coordinating co-solvents, which reveals that these are less effective in inhibiting ion pair formation. The electrolyte containing VC even increases ion pair formation in comparison to the neat electrolyte, which has been similarly observed in PEO electrolyte systems.^26^ But apart from VC, the variation in *f* is small and in the range from 61% to 85%.

Comparing the neat electrolyte at *r* = 0.25 to LiTFSI in PCL at *r* = 0.1, where the free anion fraction amounts to only 19%,^56^ the higher salt concentration promotes dissociation, which is also consistent with an increasing effective charge of Li, as shown earlier.^18^ A varied polymer end cap might also influence *f*_free_ due to the rather short polymer chain length used (*M*_n_ = 3500 g mol^−1^). Apparently, at a high salt concentration (*r* = 0.25), a comparably high dissociation is already achieved without plasticization, which is why the addition of CS only has a minor effect. Nevertheless, even with a strongly coordinating co-solvent like 15C5 in excess, where all co-solvents are expected to be coordinated by the crown ether,^26,57^ ionic dissociation is not complete, which could indicate a partly shared coordination of cations by co-solvent and anion.

In view of the variations of the diffusion coefficients and the degree of ion pairing with the type of co-solvent the nearly unaltered apparent lithium transference number in Fig. 2 is quite remarkable. In the following, we will shed light on this feature by analyzing the migration in the electric field for all constituents. To this end, we focus on the most relevant co-solvents, according to the following criteria. Sulfolane causes a relatively good dissociation and high apparent transference number as well as a low correlation of Li to the polymer, although the absolute diffusion coefficients in this electrolyte were comparably low. G4 yielded the highest diffusion coefficients and is interesting to be compared to 15C5. At last, DMSO is a candidate of moderate binding strength to lithium cations and might represent a good compromise.

### Ion migration in PCL electrolytes

**Fig. 3:**
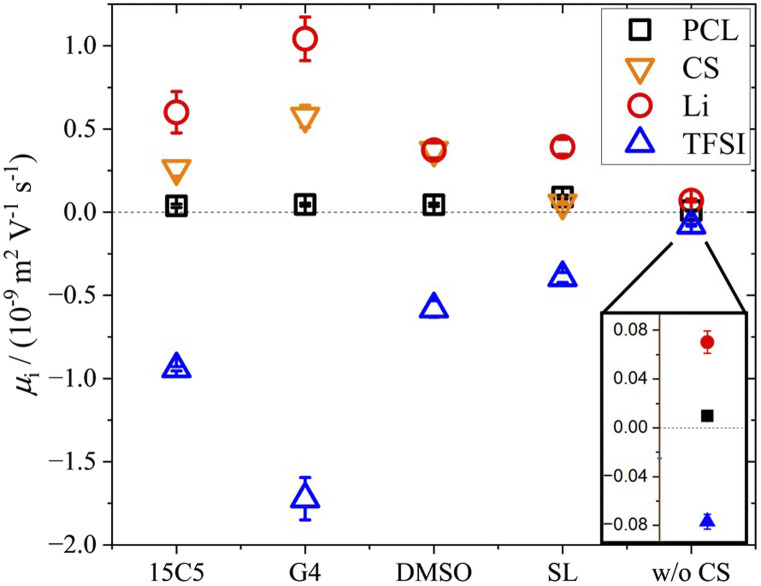
Electrophoretic mobilities *µ* at 90 °C in PCL + LiTFSI electrolytes containing either none or one of the depicted co-solvents.

**Fig. 4:**
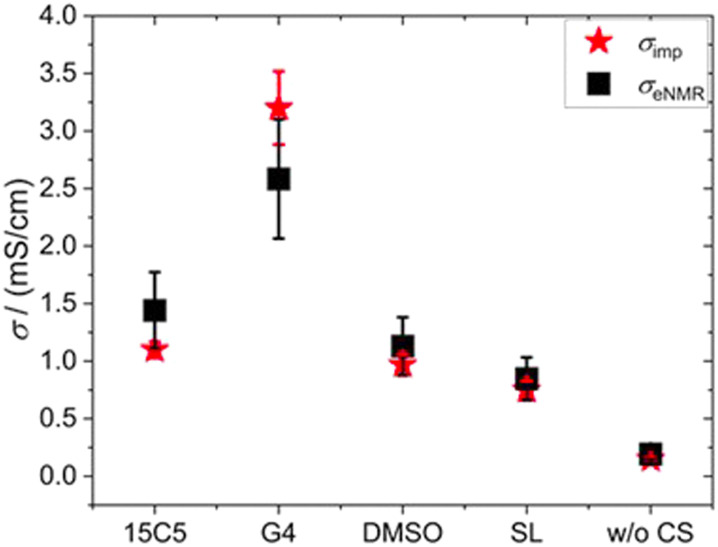
Comparison of ionic conductivities determined by impedance spectroscopy (red stars) and electrophoretic NMR (black squares). For details of the calculation see SI.

By means of electrophoretic NMR (eNMR), electrophoretic mobilities of each species were determined in the neat PCL polymer electrolyte as well as in those containing co-solvents 15C5, G4, SL and DMSO. The results are displayed in Fig. 3 and listed in Table S5, where the sign of the mobility reflects a drift direction to the positive (negative mobility) or negative electrode (positive mobility), respectively. With the electrophoretic mobilities for co-solvent containing electrolytes being about one order of magnitude larger than those in the non-plasticized system, Fig. 3 clearly evidences the effect of plasticization. This enhancement has also been observed in the diffusion coefficients in Fig. 1a and is reproduced by the ionic conductivities in Table S1. As a standard test of data quality, agreement of the latter to the sum of the partial conductivities calculated from electrophoretic mobilities is demonstrated in Fig. 4, while details of the required calculations are given in SI, eqn (S1) and (S2) and Table S1.

Interestingly, the electrophoretic mobilities reveal that negative mobilities are obtained only for the TFSI anions, while positive mobilities are obtained for all remaining components. Positive mobilities had been observed for the chains in PEO-based systems and were formerly explained by a drag by coordinated lithium ions.^58^ Although this explanation might represent part of the full picture, recent studies suggest that a significant share of the drift of non-charged components is due to the need to compensate the volume flux of large TFSI anions drifting to the positive electrode.^26,38^ At high concentrations and large anion sizes, it was found that this volume conservation constraint, motivated by the avoidance of density fluctuations, does even lead to a surprisingly fast migration of the polymer, which is close to the drift velocity of lithium cations itself.^35^ In the present electrolytes, however, the volumetric salt concentration is not as high as in the PEO electrolytes studied previously,^26^ since the PCL monomeric units are considerably larger. Therefore, a slower polymer drift is observed, which might still contain a combination of coordination and volume conservation effects.

**Fig. 5:**
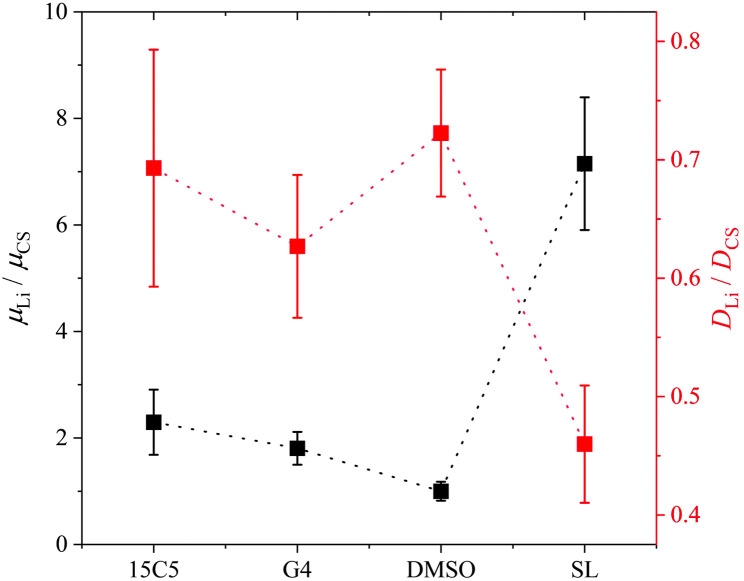
Lithium electrophoretic mobilities *µ* and diffusion coefficients *D*, each normalized to the value of the co-solvent. All data taken at 90 °C. Error bars result from error propagation.

Another interesting aspect in Fig. 3 is the difference in relative co-solvent drift velocity. For 15C5 and G4, lithium cations migrate much faster than the respective co-solvents in the electric field. Although these ether co-solvents strongly coordinate lithium cations, a significant fraction of co-solvent is not coordinated due to its excess with regard to salt concentration ([CS]/[Li] = 1.635). Since the excess CS experiences only a hydrodynamic, but not an electric force, the average CS mobility is decreased in comparison to that of the lithium cations, which are migrating in a joint Li-*co*-solvent complex. A comparison of relative mobilities and diffusion coefficients of lithium and co-solvent is presented in Fig. 5 with values listed in Table S6.

Generally, for most co-solvents the Li diffusion is about 30% slower than co-solvent diffusion, while the Li mobility is larger than the co-solvent mobility by a factor of up to two. In the case of SL, however, relative to the co-solvent, Li diffusion is reduced by about 50% while its migration is almost 8 times faster. In analogy to literature reports on highly concentrated salt-in-sulfolane electrolytes, this finding suggests an uncorrelated transport of lithium ions, in which Li^+^ can exchange coordination sites without a significant drift of co-solvent or polymer.^45^ Although in liquids the latter finding was partly challenged by the observation of significant positive correlations of sulfolane and lithium^59^ and its origin is not yet fully elucidated, clearly a very efficient translational motion of Li^+^ was concluded.^60^ Finding an extremely efficient Li migration in PCL, it could even be speculated that sulfolane somehow interacts with the rather non-polar polymer *via* van-der-Waals interactions, which immobilizes it in the polymer network. The apolar ring structure of sulfolane is also its major difference to DMSO and could explain the different behavior, contrasting their similar dielectric constants and dipole moments.^61^

**Fig. 6:**
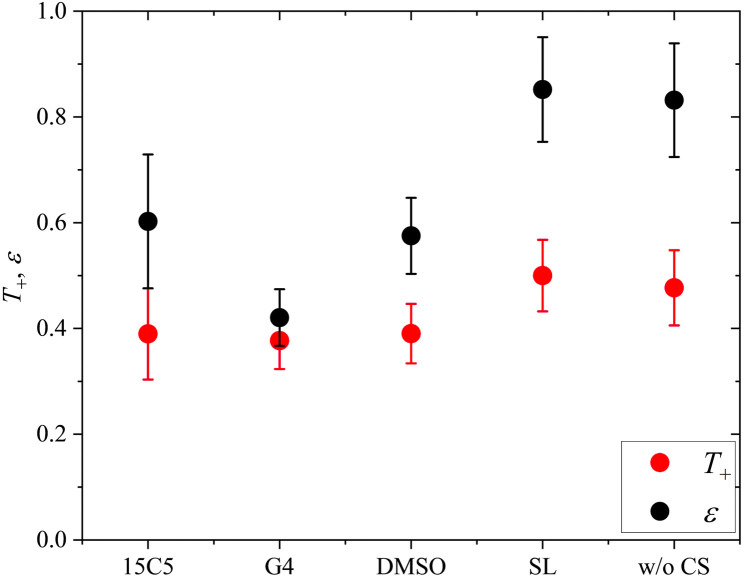
Lithium transference number *T*_+_ and ratio *ε* = *µ*_Li,eNMR_/*µ*_Li,diff_ of lithium ion mobility, measured by eNMR, divided by the mobility expected by diffusion *via* the Nernst–Einstein equation. Error bars result from error propagation.

Finally, the lithium ion transference number is calculated by the electrophoretic mobilities *via*eqn (5), and shown in Fig. 6 (values in Table S6):5
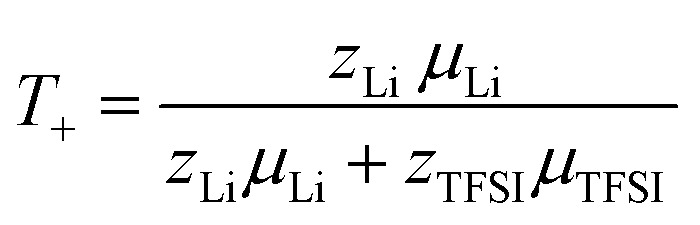


In addition, the ratio *ε* of eNMR mobility and mobility expected by diffusion *via* the Nernst–Einstein equation (eqn (6)), is displayed in Fig. 6 and listed in Table S6, together with diffusion coefficients at 90 °C.6
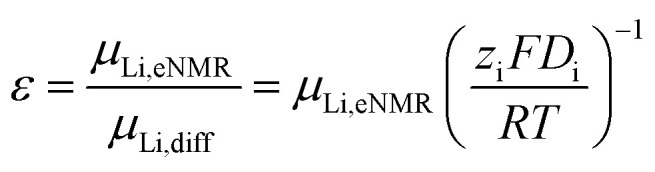


(*z*: charge number, *F*: Faraday constant, *R*: ideal gas constant, *T*: temperature) This parameter is often referred to as an “effective charge” in literature.^62,63^ It equals 1 in case of an ideal electrolyte and any deviation reflects to which extent the electric charge of an ionic constituent actually causes a drift in the electric field. A value below 1 signifies a slower electrophoretic drift than expected by diffusion *via* the Nernst–Einstein equation, for example caused by the formation of uncharged ion pairs or by other ion–ion correlations. A value above 1 might indicate correlation effects such as local volume conservation constraints or multiply charged clusters.^26,62^ Since the term “effective charge” might be misleading, we herein refer to it as a “correlation parameter” *ε*, and interpret it as a qualitative parameter indicating the role of ion correlations.

Both parameters, *T*_+_ and *ε*, are reduced in most of the electrolytes with co-solvent. In particular, the lithium ion transport efficiency, represented by *T*_+_, is clearly diminished upon the addition of 15C5, G4 or DMSO in comparison to the neat electrolyte.

*T*_+_ is not reduced by incorporation of sulfolane, where anion and cation apparently drift with the same velocity and lithium ion transport is only weakly affected by undesired correlations such as ion pair formation. Revisiting Fig. 2, a comparably high fraction of free anions is indeed observed for the SL electrolyte, agreeing to stronger lithium-sulfolane than lithium–anion interactions, as reported in literature.^59^

The reason for the decrease in transference number and correlation parameter upon addition of the remaining co-solvents has previously been discussed for the corresponding PEO-based system and was attributed to a diminished beneficial acceleration of lithium by volume conservation effects.^26^ Since the uncharged co-solvents lower the absolute charge density in the electrolytes and contribute to the compensation of the anion volume flux, they effectively lower the hydrodynamic force exerted on Li by the anions.

### Comparison of PCL and PEO electrolytes

We will now compare the co-solvent influence in PCL to previous results in PEO, as reported in a former study.^26^ In both cases, a salt to monomer ratio of *r* = [Li]/[monomer] = 0.25 was used. The higher molar fraction of co-solvent ([CS]/[Li] = 1.635), chosen in the present study, furthermore results in the same mass fraction of co-solvent as in the respective PEO system. Both systems are therefore directly comparable, but it has to be considered that the PCL monomeric units cover 7 atoms in length while an ethylene oxide unit only covers 3 atoms.

**Fig. 7:**
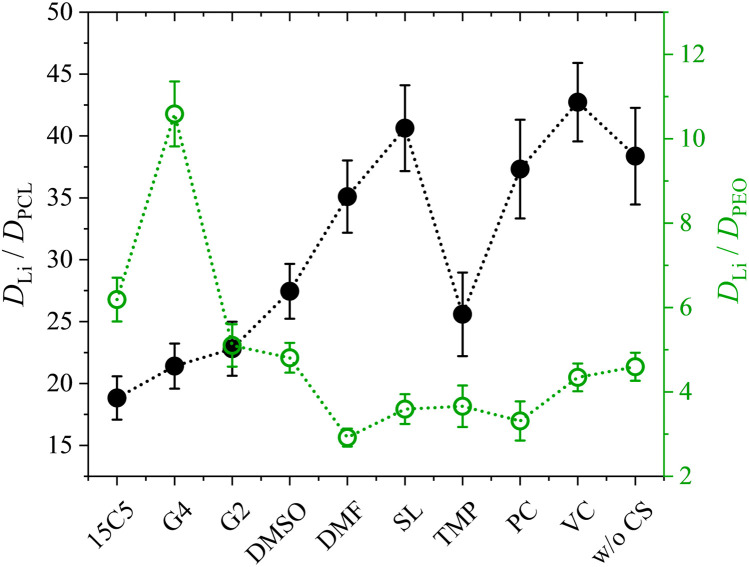
Lithium diffusion coefficients *D* at 80 °C measured by PFG-NMR normalized to the polymer diffusion in PCL + LiTFSI (this work) and PEO + LiTFSI (ref. 26) polymer electrolytes containing either none or one of the depicted co-solvents. Lines serve as guide-to-the-eye only.

**Scheme 2:**
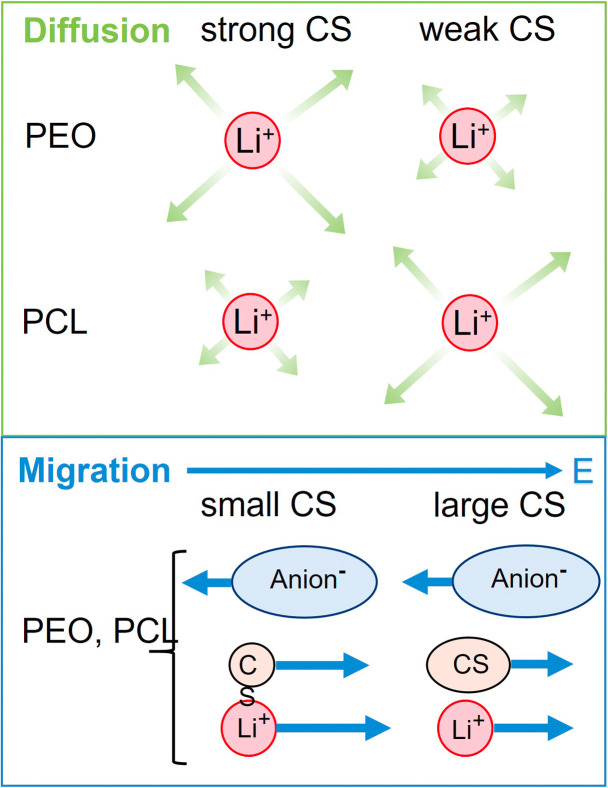
Top: simplified visualization of relative Li diffusion in PEO and PCL in dependence on co-solvent-Li^+^ coordination strength, showing opposite trends. Bottom: visualization of only minor dependence of Li migration in dependence on co-solvent size, being identical for both polymers.

Fig. 7 shows the lithium diffusion coefficients normalized to either the PCL or PEO diffusion coefficient and already reveals the most important difference of both polymer architectures. As can be seen by the different scales in Fig. 7, lithium ion diffusion in the PCL electrolytes is, in general, much faster relative to the polymer than in the PEO electrolytes. Already if no co-solvent is included, relative lithium ion diffusion is rather high in the PCL- and comparably low in the PEO-based electrolytes. Clearly, this originates from the much weaker association of lithium ions with PCL due to a weaker coordination strength.^18^ It is interesting that, while for PCL the rather weakly coordinating co-solvents provide a high level of relative lithium ion diffusion, it is the very strongly coordinating co-solvents that enhance it in PEO. As discussed above, large and strongly coordinating co-solvents appear to hinder lithium diffusion in PCL by increasing the Stokes radius and thus hampering transport through the polymer. In contrast, in PEO a strong co-solvent coordination of Li ions is needed to compete with the polymer coordination and liberate lithium ions from the polymer chains. This leads to the conclusion that coordinating co-solvents, in general, have opposing effects for both polymers: While they hinder diffusion by increasing the Stokes radius of the cationic complex in PCL, they can facilitate diffusion in PEO by a liberation from the polymer chains. This feature is shown in the upper part of Scheme 2 in a simplified manner, where the opposing effect of CS coordination strength on the relative Li diffusion is visualized.

**Fig. 8:**
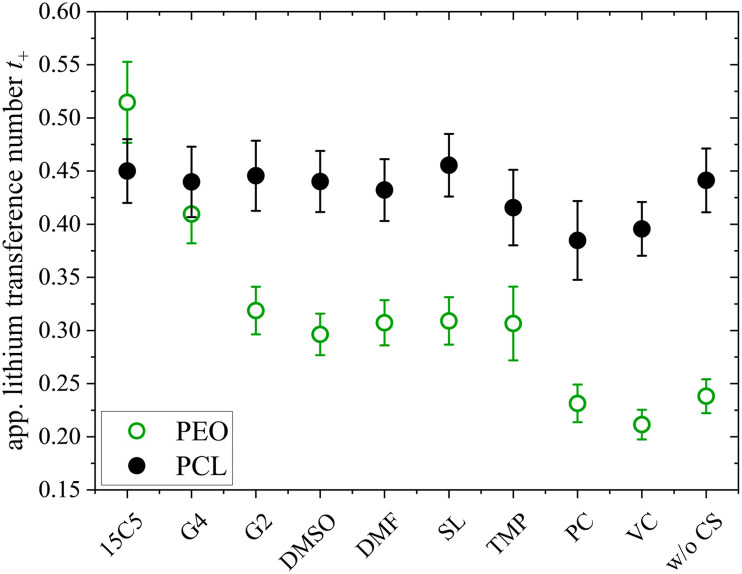
Apparent lithium transference number *t*_+_ at 80 °C determined by pulsed-field gradient NMR in PCL + LiTFSI (this work) and PEO + LiTFSI (ref. 26) electrolytes containing either none or one of the depicted co-solvents.

Another intriguing difference of both polymer architectures can be seen in Fig. 8, where the apparent lithium transference number *t*_+_, determined by PFG-NMR (eqn (4)), is displayed. While *t*_+_ strongly varies for the PEO electrolytes depending on the co-solvent coordination strength (*t*_+_ = 0.21 to 0.51), it is almost invariant on a high level for the PCL electrolytes (*t*_+_ = 0.38 to 0.45). As discussed by Rosenwinkel *et al.*, the generally higher *t*_+_ is a result of faster lithium ion diffusion due to a liberation from polymer coordination in PCL.^18^ The comparison with PEO additionally reveals that the influence of co-solvents on *t*_+_ is based on the liberation from polymer chain coordination of lithium cations. Only in PEO, where lithium cations are strongly immobilized by polymer coordination in the neat electrolyte, co-solvents can improve lithium transport by competing with the Li-polymer coordination and a similar *t*_+_ as in the PCL electrolytes can be achieved by the strongly coordinating co-solvents like 15C5 or G4.

**Fig. 9:**
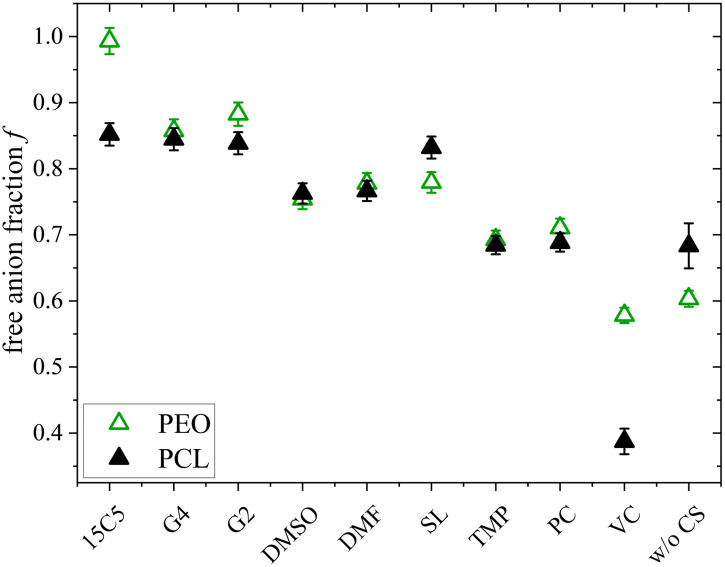
Free anion fraction *f* of PCL + LiTFSI (this work) and PEO + LiTFSI (ref. 26) electrolytes containing either none or one of the depicted co-solvents determined by Raman spectroscopy.

In this discussion we have not yet considered the role of the anion, which also contributes to eqn (4). From Raman spectroscopy the free anion ratio *f* was extracted, which is shown in Fig. 9 for both polymer architectures.

Noteworthy, the free anion fraction is almost identical for the PCL and PEO electrolytes and only differs more significantly for the neat electrolyte and the 15C5- and the VC-plasticized electrolyte. This suggests, that it almost does not matter for the anion, whether the polymer interacts strongly with the cation or not. Since by MD simulations a large dominance of a shared polymer-cation-anion coordination in non-plasticized PCL and PEO electrolytes was found,^64^ it can be concluded that it makes little difference for the anion whether this shared coordination occurs with a coordinating co-solvent or a polymer chain. Even in the neat electrolytes, the difference in dissociation is surprisingly low.

**Fig. 10:**
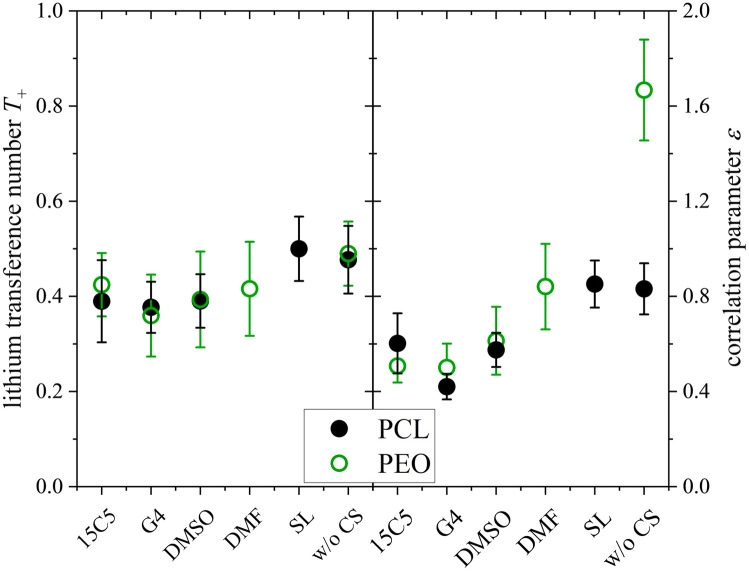
Lithium transference number *T*_+_ and correlation parameter *ε* in PCL + LiTFSI (this work) and PEO + LiTFSI (ref. 26) electrolytes containing none or one of the depicted co-solvents.

At last, the change in ion migration is of utmost importance, which is why Fig. 10 shows the lithium transference number and the correlation parameter *ε*. Surprisingly, the clearly pronounced differences of the two polymer electrolytes regarding diffusion are not seen in the lithium ion transference and migration. Especially the lithium transference number perfectly agrees for both polymers. Similar agreement is found for *ε*, only the neat electrolyte shows a major difference. In our previous work, the high value of *ε* in the neat PEO + LiTFSI electrolyte was attributed to a dominance of hydrodynamic fluxes and local volume conservation effects, initially revealed in ionic liquids.^26,38,65^ In detail, the fast drift of voluminous TFSI anions to the positive electrode leads to the acceleration of the remaining components, Li^+^ and neutral constituents, in the opposite direction to the negative electrode, see Scheme 2, bottom. Due to this strong anticorrelation, a correlation parameter far above 1 is obtained for Li. This finding was supported by the comparison of differently sized anionic species, which lead to an increase in *ε* and *T*_+_ with anion size in agreement to the dominant local volume conservation effect.^35^

Although local volume conservation does not lead to such a high *ε* in the PCL electrolyte as in the PEO case, most probably due to the lower volumetric salt concentration, the preeminence of a hydrodynamic anticorrelation can explain why only small differences are observed for both systems regarding electrophoretic migration, see illustration in Scheme 2. While varying coordination environments have a strong effect on the statistical displacements in diffusion, they do not significantly alter the impact of the anion-induced hydrodynamic flux, which affects all non-anionic components similarly. Apparently, the volume flux by the anticorrelated drift of TFSI anions is more or less evenly distributed among the remaining species, and is invariant with regard to the mutual interactions of these constituents. This is a relevant finding, since it was concluded in a former study,^18^ that in order to increase lithium ion transference, weakly coordinating polymers are superior due to a weaker immobilization of lithium ions in the polymer network. However, our results indicate that, at high salt concentration (here *r* = 0.25), volumetric effects caused by the flux of the considerably more voluminous anionic species are dominating lithium transference, making *T*_+_ effectively independent on polymer coordination strength.

## Conclusions

In conclusion, a range of co-solvents and their influence on ion transport in electrolytes comprised of PCL and LiTFSI ([Li^+^]/[monomer] = 0.25) was studied by pulsed-field gradient and electrophoretic NMR. While diffusion coefficients are effectively increased by co-solvent plasticization, it was found that especially large and strongly coordinating co-solvents hinder cation diffusion in the polymer matrix by increasing the Stokes radius of a cationic complex. Weakly coordinating co-solvents, in contrast, can enhance relative lithium ion diffusion, *D*_Li_/*D*_PCL_, more effectively. The apparent lithium ion transference number and ionic dissociation, however, are only mildly influenced by the nature of the co-solvent.

In an electric field, a positive migration of the uncharged polymer and co-solvents was observed and explained by the need to compensate the volume flux of anions, possibly aided by a coordination of lithium cations to the uncharged components inducing a drag in the electric field. Co-solvents were found to cause only a slight decrease in lithium ion transference compared to the non-plasticized electrolyte.

At last, the results obtained in the PCL electrolytes were compared to a similar system comprised of PEO and LiTFSI investigated in a former study.^26^ At that, a strongly contrasting effect of co-solvents was observed for both polymer architectures since co-solvents led to an increase of relative lithium ion diffusion, *D*_Li_/*D*_polymer_ in the PEO electrolytes by competing Li-polymer coordination, but provoked a decrease in the PCL-based systems. The trends of relative diffusivities are oppositely depending on co-solvent coordination strength, as strong coordination is favourable in PEO, while weak coordination is favourable in PCL.

In contrast to these strong trends, the migration of species is hardly affected by coordination effects. Here, the transference numbers are independent on the type of polymer and only weakly influenced by co-solvent coordination strength. The explanation for these uniform properties is the fact that instead of coordination, migration is largely dominated by hydrodynamic fluxes caused by local volume conservation. Driven by the large volume flux of the bulky anions in the electric field, all other species experience an opposite migration direction. Despite the contrasting coordination characteristics of PEO and PCL, this causes a very similar behavior of both polymeric systems under the influence of an electric field.

All in all, our results indicate that the nature of the polymer strongly alters the effect of co-solvents on species diffusion. In contrast, the choice of co-solvent was shown to be of less significance for the migration of ionic species and the Li transference number. At the present high salt concentration a dominance of correlated hydrodynamic effects outweighs effects by coordination characteristics of individual species.

As a consequence, the choice of any plasticizing co-solvent does not need to be driven by considerations of its coordination behavior. Efficient Li^+^ ion transport in terms of a high Li^+^ transference number is best achieved by a minimal compensation of anion volume flux by neutral species' volume. Therefore, small co-solvents should be favoured and the highest *T*_+_ is achieved with no co-solvent.

While our study was performed on rather low molecular weight polymers, the findings disclose general concepts concerning the balance of hydrodynamic fluxes of different species and the implications for Li^+^ transference. Generally, for higher molecular weights slower diffusion and migration is expected, however, the general trends and influences of individual species' hydrodynamic fluxes in an electric field should apply in the same way. Similarly, the general trends extracted here at 90 °C will also apply to lower temperatures in the operating range of battery cells, since both polymers have a very low *T*_g_ and above *T*_g_ the *T*-dependence of the transport parameters is governed by a monotonous VTF behavior.

Finally, in view of a conceptually different influence of co-solvent coordination strength on diffusion and migration, respectively, the use of diffusion coefficients as an indicator for Li transference, *i.e*. estimating an apparent transference number as in eqn (4), becomes even more questionable.

## Author contributions

Conceptualization, S. B. and M. S.; formal analysis, S. B.; investigation, S. B.; resources, M. S.; writing – original draft, S. B.; writing – review & editing, S. B. and M. S.; visualization, S. B.; supervision, M. S.; funding acquisition, S. B. and M. S.

## Conflicts of interest

There are no conflicts to declare.

## Supplementary Material

SC-OLF-D6SC00596A-s001

## Data Availability

The supporting data has been provided as part of the supplementary information (SI). Supplementary information: NMR spectra: Fig. S1 and S2; diffusion echo decays: Fig. S3 and S4; eNMR pulse sequence: Fig. S5; eNMR phase shifts: Fig. S6, densities and ionic conductivities: Table S1, numerical values of diffusion coefficients: Table S2, Raman spectra, their deconvolution and free anion fraction: Fig. S8 and Tables S3 and S4, numerical values of electrophoretic mobilities: Table S5, diffusion coefficients, transference numbers and correlation parameters: Fig. S7 and Table S6. See DOI: https://doi.org/10.1039/d6sc00596a.
