# Supplementary material for: Li diffusion and migration are influenced differently by co-solvents in polymer electrolytes based on poly(ε-caprolactone) and poly(ethylene oxide)
Source: Chem Sci. 2026 May 5;17(24):11984–95. doi: 10.1039/d6sc00596a (PMC13159014; doi:10.1039/d6sc00596a)
Supplement: SC-017-D6SC00596A-s001 [file SC-017-D6SC00596A-s001.pdf]

Supplementary Information (SI) for Chemical Science.
This journal is © The Royal Society of Chemistry 2026

Supporting Information for

Li diffusion and migration are influenced differently by co-solvents in polymer electrolytes based on poly(ε-caprolactone) and poly(ethylene oxide)

Simon Buyting[a,b], Monika Schönhoff[a]

[a] *Institute of Physical Chemistry, University of Münster, Corrensstr. 28/30, 48149 Münster, Germany*

[b] *International Graduate School for Battery Chemistry, Characterization, Analysis, Recycling and Application (BACCARA), University of Münster, 48149 Münster, Germany*

Email: s.buyting@uni-muenster.de, schoenho@uni-muenster.de

Table of Contents

1. NMR spectra ... S-2

2. Diffusion NMR – Raw data ... S-4

3. Electrophoretic NMR – Raw data ... S-6

   3.1 Comparison of ionic conductivities ... S-7

4. Diffusion NMR - Results ... S-8

5. Raman Spectroscopy ... S-9

6. Electrophoretic NMR – Results ... S-11

References ... S-11

1. NMR spectra

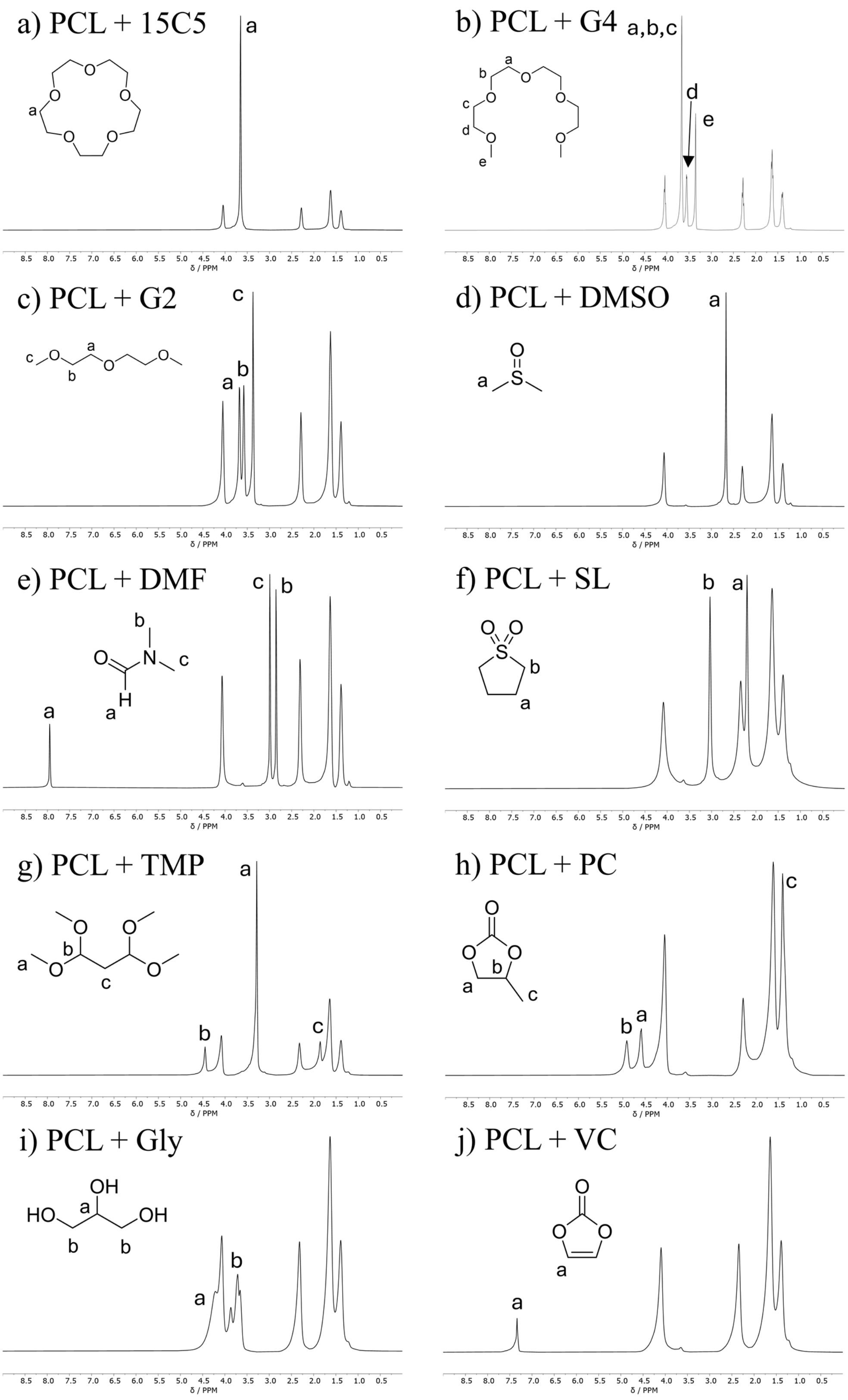

Figure S1. Assignment of co-solvent resonances in ^{1}H NMR spectra of PCL+LiTFSI electrolytes containing different co-solvents at 80 °C. Spectra were referenced according to [1].

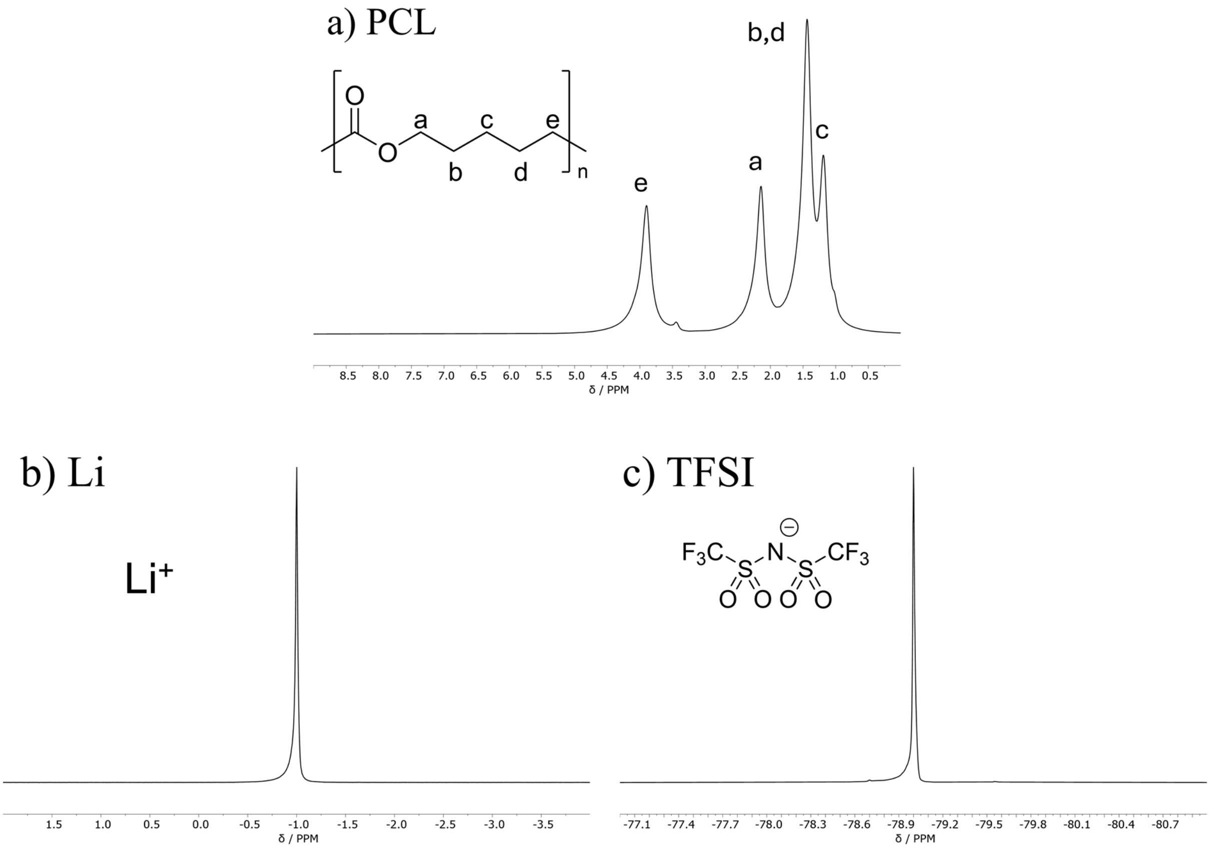

Figure S2. Exemplary a) ^{1}H, b) ^{7}Li and c) ^{19}F spectra of the PCL+LiTFSI electrolyte at 80 °C. Spectra were referenced according to [2].

2. Diffusion NMR – Raw data

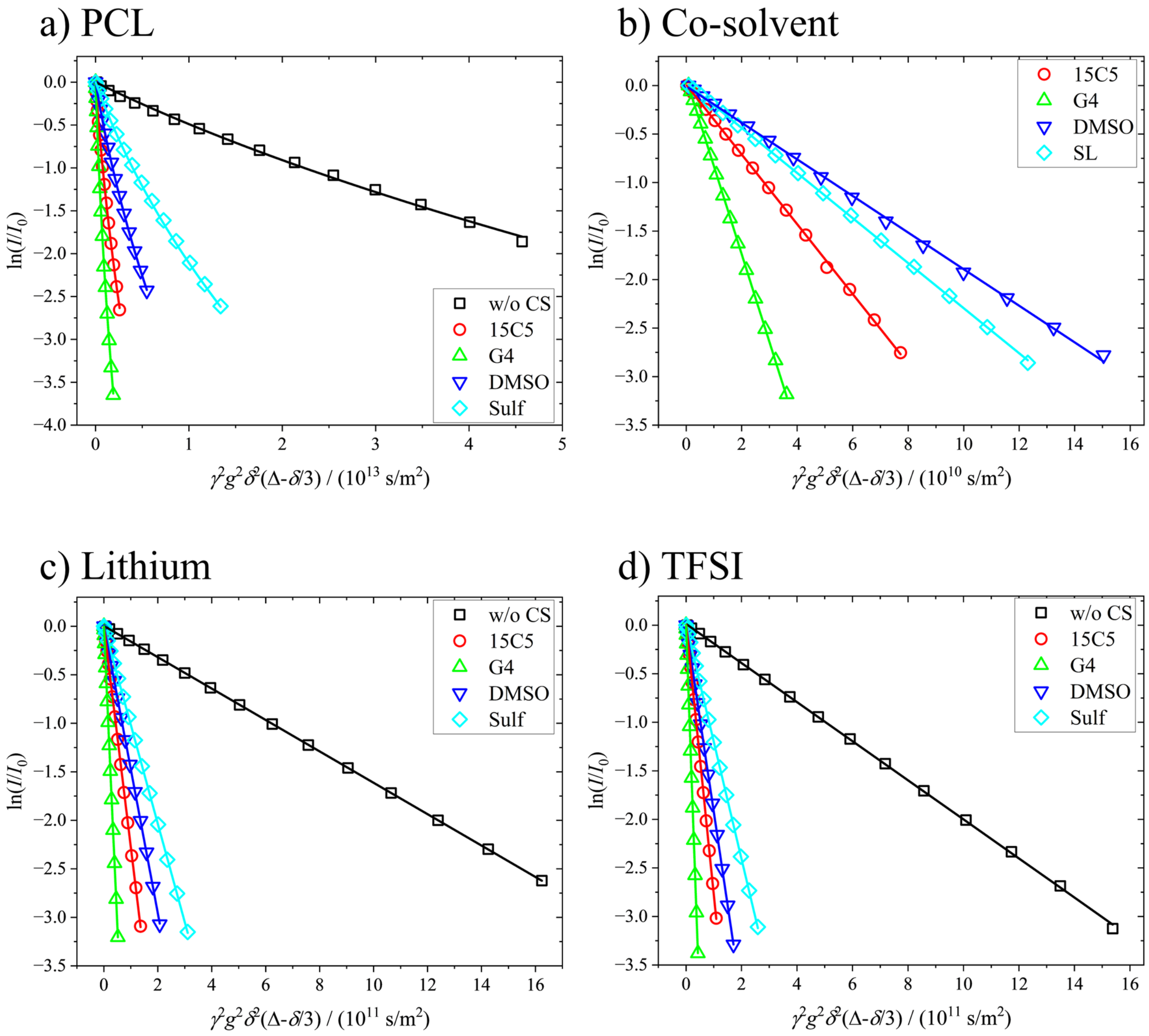

Figure S3. Exemplary Stejskal-Tanner plots of a) PCL (^{1}H), b) co-solvent (^{1}H), c) lithium cations (^{7}Li) and d) TFSI anions (^{19}F) at 80 °C. The legend specifies the respective co-solvent in the electrolyte. For the polymer PCL (a), a log-normal distribution of *D* was fitted to account for its polydispersity. The intensity loss of the remaining components (b-c) was linearly fitted.

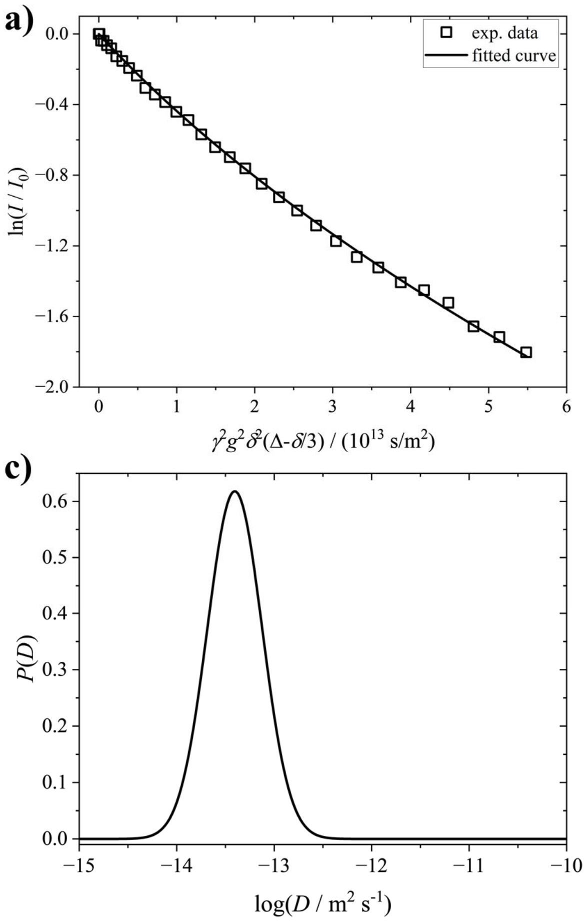

Figure S4. Exemplary Stejskal-Tanner plots (a-b) and diffusion coefficient distributions $P(D)$ (c-d) of the neat PCL+LiTFSI electrolyte (a,c) and of the electrolyte containing 15C5 (b,d). To extract $P(D)$, the signal attenuation curves (a-b) were fitted by a log-normal distribution of diffusion coefficients resulting from the polymer's polydispersity.

3. Electrophoretic NMR – Raw data

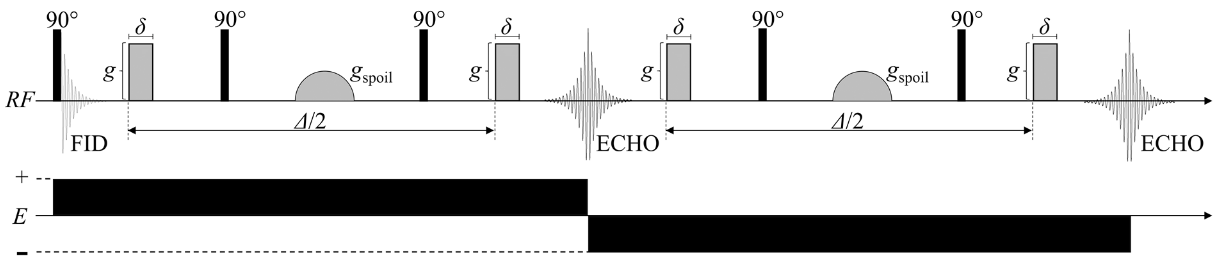

Figure S5. Double stimulated echo (DSTE) pulse sequence, accompanied by two electric field pulses of same strength but opposite polarity as employed to obtain electrophoretic mobilities by electrophoretic NMR.

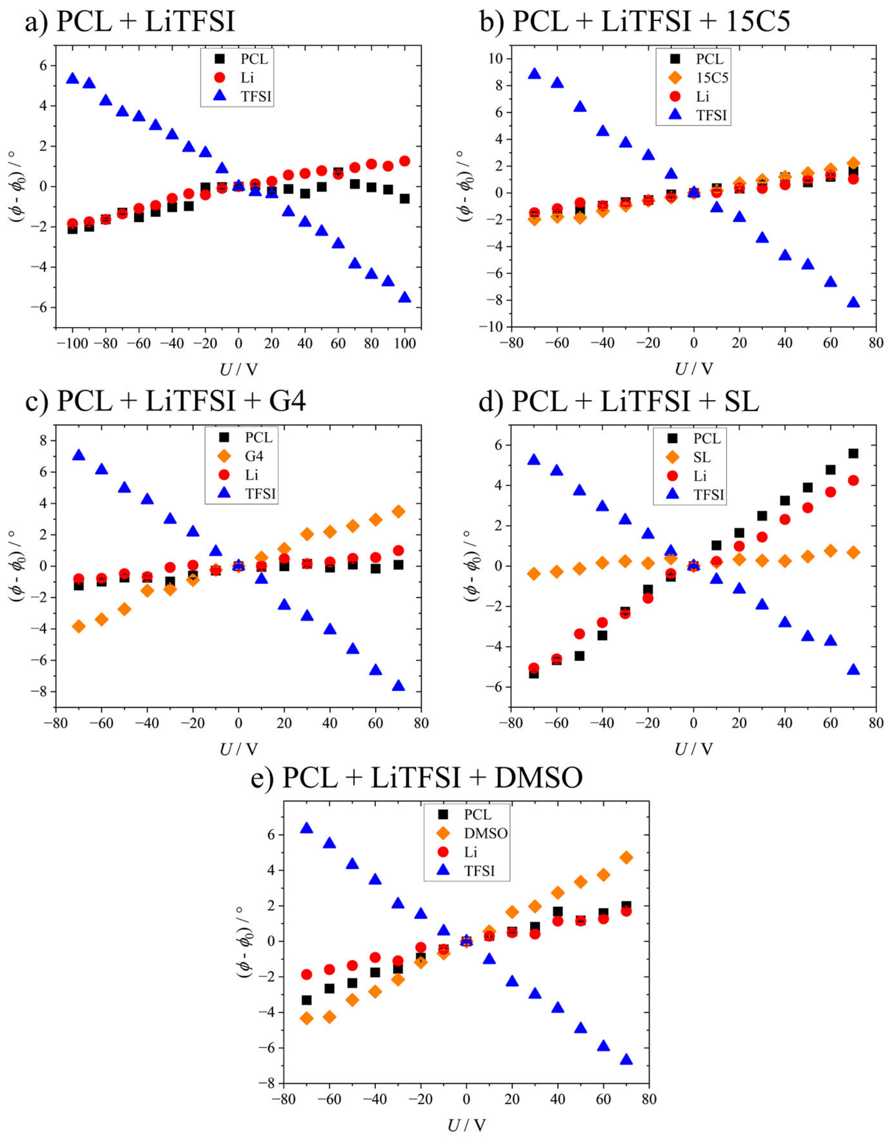

Figure S6. Exemplary phase shift plots of PCL+LiTFSI electrolytes containing either no (a) or one of the co-solvents 15C5 (b), G4 (c), SL (d) or DMSO (e). Error bars mostly lie within the data points and result from the fitting procedure. Please note that similar phase shift magnitudes do not necessarily imply similar ionic mobilities due to variations in gradient strength and duration.

3.1 Comparison of ionic conductivities

A consistency check of the ionic mobilities obtained by electrophoretic NMR (eNMR) is performed by calculating the total ionic conductivity σ_{eNMR} by the summed partial conductivities, and comparison to the total ionic conductivity as obtained from impedance spectroscopy.

$$\sigma_{\text{eNMR}} = \sum_i \sigma_i = \sum_i z_i\, c_i\, \mu_i\, F \qquad \text{(S1)}$$

At that, the partial conductivity σ_i results from the charge number z_i, salt concentration c_i, electrophoretic mobility μ_i and Faraday constant F. To determine the salt concentration via Eq. S2, sample densities at 90 °C were measured using a helium-operated *AccuPyc II 1340* gas pycnometer (*Micromeritics*, Norcross GA, USA). For that purpose, the instrument was calibrated using a standard metal sphere (*Micromeritics*) of defined volume and samples were measured in standardized 1 cm^3 aluminum crucibles containing at least 1 g of sample. The results are listed in Table S1.

$$c_{\text{Li}} = \frac{n_{\text{Li}} \cdot \rho \cdot 10^3}{m_{\text{ges}}} \qquad \text{(S2)}$$

Table S1: Electrolyte densities and ionic conductivities determined by impedance spectroscopy of either the neat PCL+LiTFSI electrolyte or the electrolytes containing the depicted co-solvents. A relative error of 10% should be considered.

**Co-solvent**	-	15C5	G4	SL	DMSO
**ρ / (g/cm^3)**	1.21	1.15	1.10	1.16	1.13
**σ / (mS/cm)**	0.19	1.29	3.20	0.93	1.12

Conductivities calculated by equ. S1 are presented in Figure 4, where they are also compared to conductivities measure by impedance spectroscopy.

4. Diffusion NMR - Results

Table S2. Diffusion coefficients of PCL+LiTFSI electrolytes containing different co-solvents and of the neat electrolyte at 80 °C obtained by PFG-NMR. The error represents the standard error of at least 4 different measurements and an assumed instrumental error of 5%.

Co-solvent	D_{PCL} / (10^{-13} m²/s)	D_{CS} / (10^{-11} m²/s)	D_{Li} / (10^{-11} m²/s)	D_{TFSI} / (10^{-11} m²/s)
15C5	12.2 ± 0.9	3.5 ± 0.2	2.3 ± 0.1	2.8 ± 0.2
G4	28 ± 2	10.1 ± 0.6	6.1 ± 0.4	7.7 ± 0.5
G2	14 ± 1	6.8 ± 0.4	3.1 ± 0.2	3.9 ± 0.2
DMSO	5.4 ± 0.3	2.1 ± 0.1	1.48 ± 0.08	1.9 ± 0.1
DMF	5.5 ± 0.3	3.3 ± 0.2	1.9 ± 0.1	2.5 ± 0.1
SL	2.5 ± 0.2	2.4 ± 0.1	1.01 ± 0.05	1.21 ± 0.07
TMP	3.9 ± 0.4	5.7 ± 0.4	1.01 ± 0.07	1.4 ± 0.1
PC	3.2 ± 0.2	5.2 ± 0.6	0.64 ± 0.03	1.9 ± 0.2
VC	1.50 ± 0.08	7.2 ± 0.4	0.64 ± 0.03	0.98 ± 0.05
GL	2.6 ± 0.1	1.5 ± 0.2	1.06 ± 0.06	0.98 ± 0.06
-	0.42 ± 0.04	-	0.161 ± 0.009	0.20 ± 0.01

Table S3. Binding constants of co-solvents to lithium ions taken from [3], apparent lithium ion transference number derived from anion and cation diffusion coefficients and free anion fraction, analyzed by Raman spectroscopy.

Co-solvent	K_a / M^{-1}	t_+	'free' anion fraction
15C5	28000 ± 5000	0.45 ± 0.03	0.85 ± 0.04
G4	74 ± 4	0.44 ± 0.03	0.84 ± 0.04
G2	11 ± 1	0.45 ± 0.03	0.84 ± 0.04
DMSO	6.0 ± 0.2	0.44 ± 0.03	0.76 ± 0.04
DMF	4.36 ± 0.07	0.43 ± 0.03	0.77 ± 0.04
SL	(8.15 ± 0.08) $\cdot 10^{-2}$	0.46 ± 0.03	0.83 ± 0.04
TMP	(7.8 ± 0.2) $\cdot 10^{-2}$	0.42 ± 0.04	0.68 ± 0.03
PC	(4.38 ± 0.09) $\cdot 10^{-2}$	0.38 ± 0.04	0.69 ± 0.03
VC	(5.14 ± 0.02) $\cdot 10^{-6}$	0.40 ± 0.03	0.39 ± 0.02
GL	-	0.52 ± 0.04	0.61 ± 0.03
-	-	0.44 ± 0.03	0.68 ± 0.03

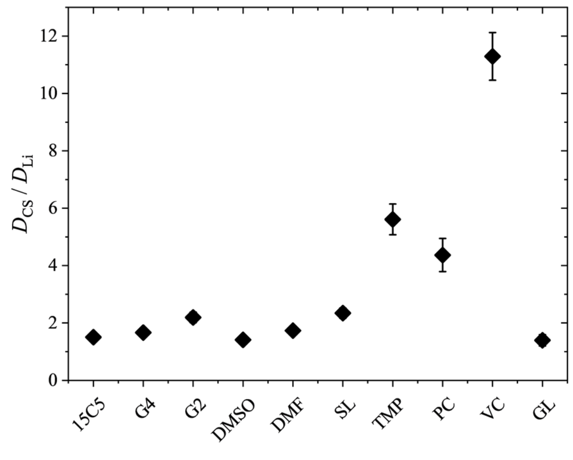

Figure S7. Co-solvent diffusion coefficient normalized to the lithium diffusion coefficient in electrolytes containing PCL+LiTFSI and different co-solvents. Error bars result from error propagation. The co-solvent coordination strength to lithium cations, except for glycerol, decreases from left to right.

5. Raman Spectroscopy

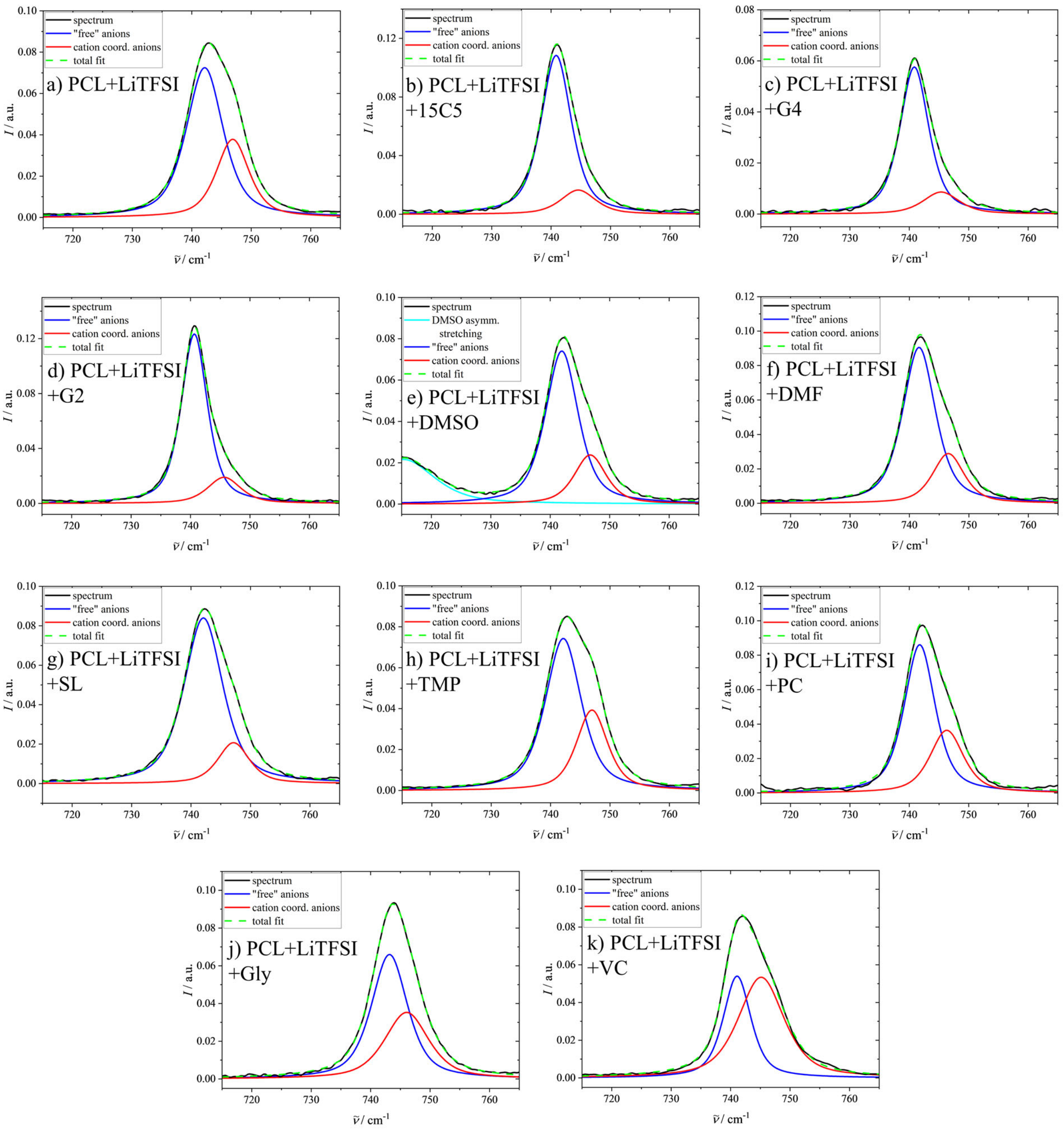

Figure S8. Raman spectra of PCL+LiTFSI electrolytes containing different co-solvents. Bands of free and cation-coordinated anions were deconvoluted assuming a band shape of 60% Lorentz/40% Gauss. In case of DMSO-containing electrolyte, the slightly overlapping DMSO asymmetric stretching mode was considered as a separate peak.

Table S4. Parameters of the deconvolution of the Raman bands. Two bands, representing free and bound TFSI, *rsp.*, were fitted by fixed band shapes of 60% Lorentz/40% Gauß. Band positions *p*, amplitudes *a* and widths *w* were free parameters, adjusted to the individual spectra. The band integral (area A) is used to calculate the free anion fraction as described in context of Figure 2.

Co-solvent	p_{free} / cm^{-1}	a_{free} /a.u.	w_{free} / cm^{-1}	A_{free} / a.u.	p_{bound} / cm^{-1}	a_{bound} / a.u.	w_{bound} / cm^{-1}	A_{bound} / a.u.	f_{free}
15C5	740.8	0.036	6.07	0.28	744.6	0.005	6.99	0.05	0.85 ± 0.04
G4	740.8	0.032	5.77	0.24	745.4	0.005	7.22	0.04	0.84 ± 0.04
G2	740.6	0.049	5.20	0.33	745.6	0.008	6.60	0.06	0.84 ± 0.04
DMSO	741.9	0.038	6.55	0.32	746.7	0.012	6.37	0.10	0.76 ± 0.04
DMF	741.6	0.042	6.56	0.36	746.5	0.014	6.27	0.11	0.77 ± 0.04
SL	742.1	0.052	7.77	0.51	747.2	0.013	6.29	0.10	0.83 ± 0.04
TMP	742.1	0.114	7.19	1.05	747.0	0.060	6.24	0.49	0.68 ± 0.03
PC	741.8	0.046	6.18	0.36	746.3	0.019	6.67	0.16	0.69 ± 0.03
VC	741.1	0.074	5.51	0.53	745.1	0.074	9.14	0.84	0.39 ± 0.02
GL	743.2	0.026	7.22	0.24	746.1	0.014	8.79	0.16	0.61 ± 0.03
No CS	742.9	0.085	7.37	1.54	746.9	0.085	6.52	0.71	0.68 ± 0.03

6. Electrophoretic NMR – Results

Table S5. Electrophoretic mobility of each constituent within electrolytes comprised of PCL, LiTFSI and either none or one of the depicted co-solvents.

Co-solvent	μ_{PCL} / (10^{-11} m^2 V^{-1} s^{-1})	μ_{CS} / (10^{-10} m^2 V^{-1} s^{-1})	μ_{Li} / (10^{-10} m^2 V^{-1} s^{-1})	μ_{TFSI} / (10^{-10} m^2 V^{-1} s^{-1})
15C5	4 ± 1	2.6 ± 0.4	6 ± 1	-9.4 ± 0.1
G4	4.6 ± 0.8	5.8 ± 0.7	10 ± 1	-17 ± 1
DMSO	4.5 ± 0.9	3.7 ± 0.5	3.7 ± 0.5	-5.8 ± 0.5
SL	9.1 ± 1.1	0.55 ± 0.07	3.9 ± 0.5	-3.9 ± 0.3
w/o CS	1.0 ± 0.3	-	0.70 ± 0.09	-0.77 ± 0.06

Table S6. Lithium ion diffusion coefficient at 90°C; Li diffusion coefficient and electrophoretic mobility, both at 90°C, normalized to the co-solvent; as well as lithium ion transference number calculated by electrophoretic mobilities and the correlation parameter *ε*.

Co-solvent	D_{Li} / (10^{-11} m^2/s)	D_{Li} / D_{CS}	μ_{Li} / μ_{CS}	T_+	$\varepsilon = \mu_{Li,eNMR} / \mu_{Li,diff}$
15C5	3.1 ± 0.3	0.7 ± 0.1	2.3 ± 0.6	0.39 ± 0.09	0.6 ± 0.1
G4	7.8 ± 0.5	0.63 ± 0.06	1.7 ± 0.3	0.37 ± 0.05	0.40 ± 0.05
DMSO	2.0 ± 0.1	0.72 ± 0.05	1.0 ± 0.2	0.39 ± 0.06	0.58 ± 0.07
SL	1.44 ± 0.07	0.46 ± 0.05	7 ± 1	0.50 ± 0.07	0.9 ± 0.1
w/o CS	0.26 ± 0.01	-	-	0.48 ± 0.07	0.8 ± 0.1

References

1. Su, J.; Xu, G.; Dong, B.; Yang, R.; Sun, H.; Wang, Q., Closed-loop chemical recycling of poly(ε-caprolactone) by tuning reaction parameters. *Polym. Chem.* **2022,** *13* (41), 5897-5904.
2. Wang, E.; Jónsson, E.; Grey, C. P., NMR Methodology for Measuring Dissolved O2 and Transport in Lithium–Air Batteries. *J. Phys. Chem. C* **2023,** *127* (21), 10001-10011.
3. Buyting, S.; Schönhoff, M., Influence of various co-solvents on ion transport in concentrated poly-(ethylene oxide)-based polymer electrolytes. *Electrochim. Acta* **2025,** *519*, 145839.
